# Freestyle multiple-perforator Island flaps for reconstruction of large dorsolumbar defects: A retrospective case series

**DOI:** 10.1016/j.jpra.2026.05.050

**Published:** 2026-06-06

**Authors:** Oswaldo J. Gómez, William D. Balaguera, David F. Duque, Diana C. Martínez, Luz E. Rueda, Liceth L. Patarroyo, Angélica M. Bayona, Sofia E. Muñoz

**Affiliations:** a Plastic Surgery Unit, Universidad Nacional de Colombia, Bogotá, Colombia; b Faculty of Medicine, Universidad Nacional de Colombia, Bogotá, Colombia; c Plastic Surgery Unit, Clínica Infantil Santa María del Lago, Bogotá, Colombia; d Clínica Universitaria Colombia, Bogotá, Colombia; e Faculty of Medicine, Fundación Universitaria de Ciencias de la Salud, Bogotá, Colombia

**Keywords:** Dorsolumbar defects, Island flaps, Freestyle flaps, Keystone flaps, Pedicular area

## Abstract

**Introduction:**

Keystone-type fasciocutaneous flaps, derived from the angiosome concept, are island flaps that have demonstrated versatility and reliability in the reconstruction of multiple anatomical regions. However, evidence regarding their use in large dorsolumbar soft-tissue defects remains limited. This study describes clinical outcomes and explores flap design parameters, particularly pedicular area proportions, in this challenging anatomical region.

**Materials and methods:**

A retrospective, non-comparative case series was conducted through medical record review of consecutive patients treated between 2021 and 2023. Patients presenting with large soft-tissue defects of the dorsolumbar region who underwent reconstruction using freestyle multiple-perforator island flaps were included. Sociodemographic data, defect characteristics, flap design variables, and postoperative outcomes were extracted and analyzed descriptively.

**Results:**

Twenty-one flaps were performed in 21 patients, with a mean age of 35 years. The mean defect size was 120 cm² (range, 12–353 cm²), and the mean flap size was 283 cm² (range, 27–589 cm²). The mean pedicular area represented 15% of total flap surface. During follow-up, four flaps developed minor wound dehiscence managed conservatively, and one flap presented transient venous congestion. A single case of partial flap loss was observed, with no cases of total flap loss. Notably, flap viability was preserved even in cases with pedicular areas as low as 10%.

**Conclusion:**

In this retrospective series, freestyle multiple-perforator island flaps provided reliable coverage with low complication rates, even with pedicular areas as low as 10–15%, suggesting greater design flexibility than traditionally assumed. These findings support their role as a practical locoregional reconstructive option with limited donor-site morbidity and reduced technical complexity. Further comparative studies are required to validate these observations.

## Introduction

Soft tissue defects in the dorsolumbar region can result from various etiologies, including infections, trauma, oncologic resections, pressure ulcers, and burns. Traditionally, reconstructive strategies have favored the use of muscle or musculocutaneous flaps, relying on the region’s rich vascular supply. More recently, fasciocutaneous and perforator flaps have emerged as effective alternatives that preserve muscle function.^1^

Advancements in cutaneous vascular studies have led to the development of key concepts such as the perforasome, which, alongside detailed anatomical descriptions, has supported the expansion of perforator-based flap techniques. However, these approaches often require meticulous dissection, may be technically demanding, and can be associated with donor-site morbidity.^2^

In 2003, Felix Behan introduced the Keystone fasciocutaneous flap, describing a trapezoidal island flap based on the **angiosome concept**. This technique involves subfascial dissection and elevation of up to two-thirds of the flap. Multiple case series have demonstrated its versatility, reliability, and efficiency across a wide range of reconstructive scenarios.^3^, ^4^, ^5^, ^6^ Subsequent refinements have incorporated the concept of the pedicular area, defined as the portion of the flap that remains attached to its bed. This area preserves a network of subfascial and subdermal vascular plexuses that function as a hemodynamic unit, enabling perfusion through the recruitment of choke vessels.^7^

Despite these advances, evidence regarding the use of island neurovascular and keystone-type flaps in large dorsolumbar defects remains limited, particularly with respect to optimal flap design parameters, including pedicular area proportions, and associated complication profiles in this anatomically demanding region.

The aim of this study is to describe clinical outcomes and evaluate flap design characteristics, with particular emphasis on pedicular area dimensions, in patients undergoing reconstruction of large dorsolumbar defects using freestyle multiple-perforator island flaps.

## Materials and methods

A retrospective, non-comparative case series was conducted including consecutive patients treated between 2021 and 2023, based on the clinical practice of the primary authors. Patients presenting with large soft tissue defects of the dorsolumbar region who underwent reconstruction using freestyle multiple-perforator island flaps were identified through medical record review. Both oncologic and non-oncologic defects were included, as well as immediate and delayed reconstructions.

Patients were excluded if reconstruction was performed using alternative techniques, if clinical data were incomplete, or if postoperative follow-up information was unavailable.

Data were extracted from medical records and included sociodemographic characteristics, defect etiology, defect size, flap design parameters (including flap size and pedicular area proportion), operative time, length of hospital stay, and postoperative outcomes. Potential confounding variables, such as prior radiotherapy and defect etiology, were also recorded and analyzed descriptively.

All procedures were performed by the same surgical team following standardized operative principles. To reduce selection bias, all eligible consecutive patients treated during the study period were included. Patients were followed in the outpatient clinic according to routine clinical practice, and available photographic documentation was reviewed.

The primary outcome was flap viability. Secondary outcomes included postoperative complications, categorized as minor (not requiring surgical intervention) and major (requiring reintervention or posing a life-threatening risk).

This study is reported in accordance with the STROBE Statement guidelines. Institutional ethical approval for retrospective data review was obtained, and the study was conducted in accordance with the Declaration of Helsinki. Data were anonymized prior to analysis.

## Surgical technique

**Fig. 1:**
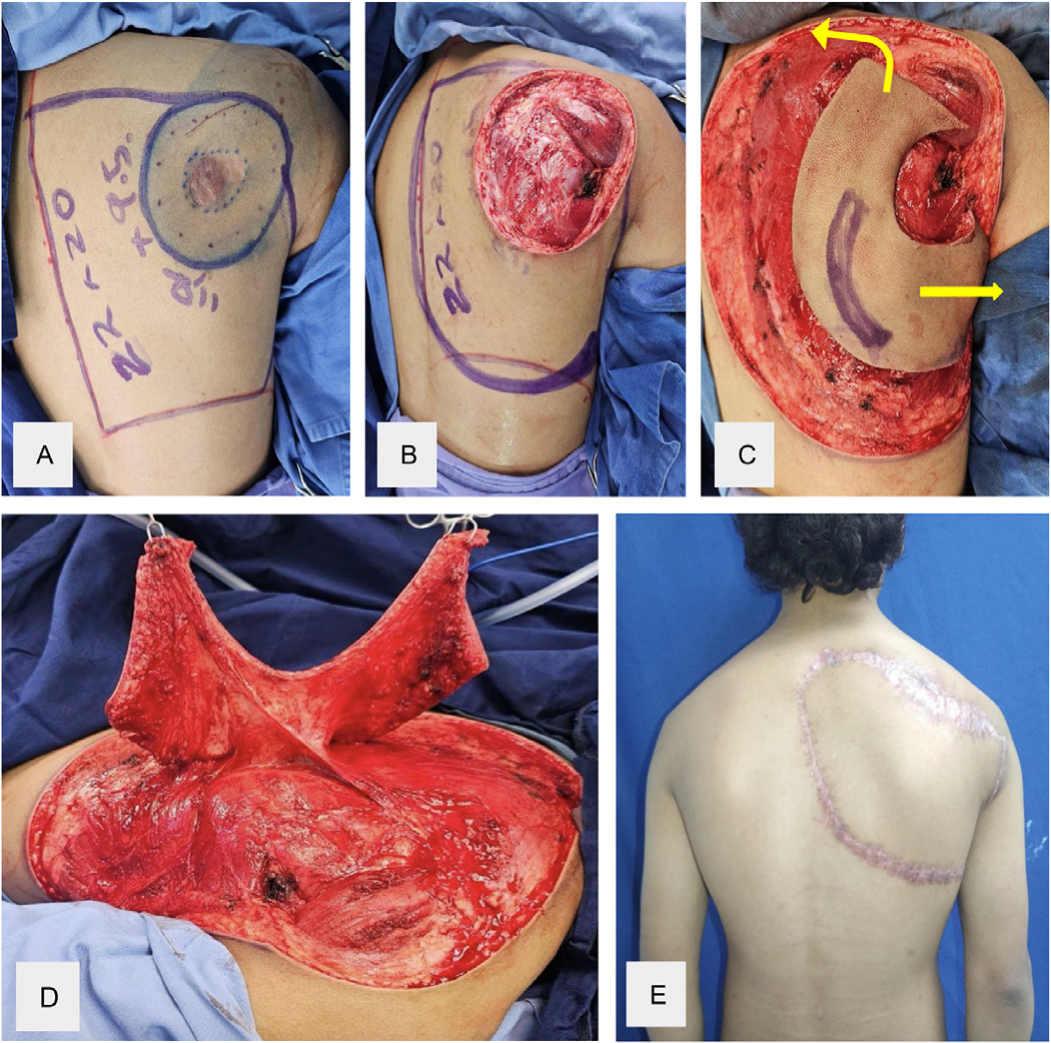
A) Dermatofibrosarcoma protuberans, B) 106 cm² coverage defect, C) Flap design and pedicular area. Horizontal distribution of the flap skin for defect coverage. D) Lateral view of the flap showing the pedicular area. (334 cm² flap with a 10% pedicular area) E) Outcome at 3 months.

We **assessed** the size and location of the defect, identifying perilesional areas with greater skin laxity and incorporating potential V-Y advancement closure patterns into the design when necessary. The mobility range of the flap is directly proportional to its size and inversely proportional to the size of the defect, particularly in cases of irradiated tissue or scar sequelae due to loss of tissue elasticity (Fig. 1).

All flaps were designed strictly as island flaps, and dissection was performed in the subfascial plane. No imaging studies or perforator mapping were performed prior to surgery. Flap elevation was conducted to achieve sufficient mobility for complete defect coverage, which was continuously tested during dissection. Pedicular areas as small as 10% of the flap surface were positioned whenever possible over the so-called "hot spots" widely described in the literature.^4^^,^^8^^,^^9^ (Fig. 1).

**Fig. 2:**
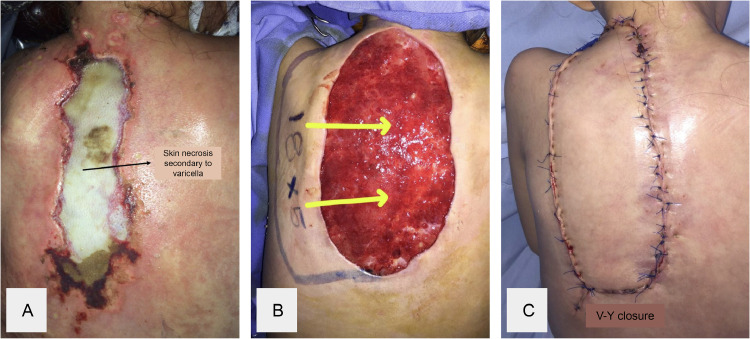
A) Skin necrosis, B) Coverage defect measuring 141 cm² after multiple debridements and negative-pressure wound therapy, horizontal advancement flap with a surface area of 102 cm², C) Follow-up at 2 weeks post-surgery.

Primary closure was achieved in all cases, avoiding layered closure in order not to compromise peripheral flap perfusion. Only skin closure was performed using staples or nylon sutures (Fig. 2).

## Results

A total of 21 flaps were performed in 21 patients (12 men and 9 women), with a mean age of 35 years. The etiology of the defect was oncologic in 13 patients and non-oncologic in 8 patients (Table 1). Fourteen flaps were used in the dorsal region, while seven were applied to the lumbosacral region. The reconstruction was immediate in 19 cases and delayed in 2 cases due to infection. Seven patients had a history of radiotherapy, which made flap dissection and mobilization difficult due to scar contractures and tissue elasticity loss.

**Table 1:** Patient clinical characteristics.

Clinical features	
Characteristics	Patient number
**Gender**	
Male	12
Female	9
**Etiology**	
Squamous cell carcinoma	4
Dermatofibrosarcoma Protuberans	4
Sarcoma	3
Meningomyelocele	2
Congenital melanocytic nevus	2
Pressure ulcers	2
Cutaneous Necrosis	2
Basal cell carcinoma	2
**Reconstruction type**	
Immediate	19
Delayed	2
**Previous radiotherapy**	
YES	7
NO	14

A total of 21 flaps were performed, with an average defect size of 120 cm² (range: 12–353 cm²). Flaps were custom-designed according to defect characteristics, with an average flap size of 283 cm² (range: 27–589 cm²). A flap-to-defect ratio of up to 5:1 was achieved, with 13 flaps undergoing primary closure using an omega pattern and 8 with longitudinal advancement. The mean pedicular area was 15% (range: 10–30%). The mean operative time for reconstruction was 142 min(range: 110–170 min), and the mean hospital stay was 3 days (range: 1–12 days) from the time of reconstruction to hospital discharge.

Follow-up was conducted for an average of 21 months (range: 2–48 months). Complications were classified as minor (not requiring surgical reintervention and not life-threatening) and major (requiring reintervention or posing a life-threatening risk) (see Table 2).

**Table 2:** Flap characteristics and postoperative outcomes.

Flap characteristics and outcomes		
Characteristics	Median (cm2)	Range (cm2)
**Defect size**	120	12–353
**Flap size**	283	27 - 589
	**Median (cm2)**	**Range (cm2)**
**Pedicular area**	15	10 - 30
	**Patient number**	
**Flap advancement type**		
Omega	13	
Longitudinal	8	
**Minor complications**		
Minor dehiscences	4	
Surgical site infection	0	
Venous congestion	1	
Partial Flap loss	1	
**Major complications**		
Total flap loss	0	
Hematomas or major bleeding	0	
Major dehiscences	0	
**Characteristics**	**Median**	**Range**
**Surgical time**	142 min	110 - 170 min
**Hospital stay**	3 days	1 - 12 days
**Follow up**	21 months	2 - 48 months

Among the cases, 4 flaps developed minor dehiscence, which resolved with conservative wound care without the need for additional surgical intervention. One flap experienced venous congestion in the immediate postoperative period, which resolved spontaneously. A single case of partial flap loss was observed, with no instances of total flap loss or major bleeding events**.** In one patient with an initial oncologic diagnosis, tumor recurrence was documented during postoperative follow-up.

## Discussion

**Fig. 3:**
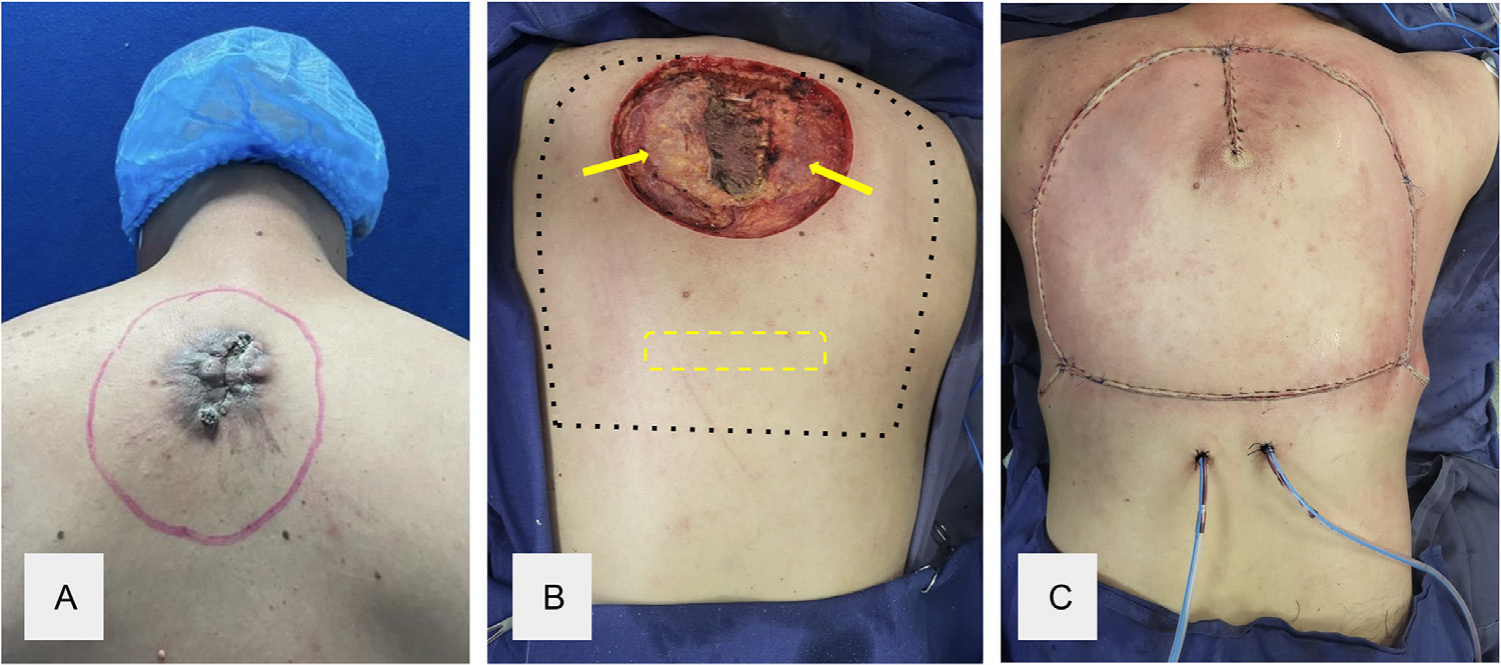
A) Nodular melanoma, B) Coverage defect measuring 225 cm², omega-shaped flap measuring 451 cm², (yellow marking represents the pedicular area - 15%), C) Immediate postoperative result, V–Y advancement closure of the inferior coverage defect.

freestyle multiple-perforator island flaps have been widely described as reliable options for reconstruction of soft-tissue defects across multiple anatomical regions.^3^, ^4^, ^5^^,^^8^^,^^9^ Their main advantages include the “like-to-like” tissue replacement inherent to locoregional flaps, preservation of underlying muscle function, and avoidance of complex microsurgical techniques. In the context of large dorsolumbar defects, these characteristics are particularly relevant, as reconstruction in this region is often limited by defect size, tissue availability, and patient comorbidities.^10^, ^11^, ^12^ (Fig. 3)

In the present series, freestyle multiple-perforator island flaps provided consistent and reliable coverage for large dorsolumbar defects, with no total flap loss and only one case of partial flap loss, and a low rate of minor complications.^3^^–^^6^

A relevant finding of this study is the observation that flap viability was preserved even with relatively small pedicular areas, reaching values as low as 10% of the total flap surface. This supports the concept that perfusion in these flaps is not solely dependent on a single dominant perforator but rather on a complex network of subdermal and subfascial vascular plexuses that function as an integrated hemodynamic unit.^13^^,^^14^ The ability to safely reduce the pedicular area may allow for greater flap mobility and flexibility in design, which is particularly advantageous in large defects or in previously irradiated tissues.

**Fig. 4:**
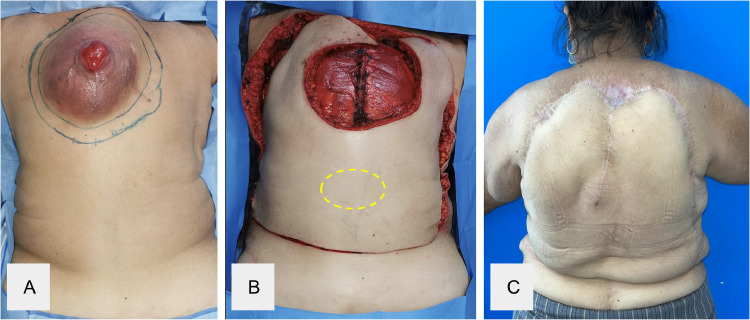
A) Dermatofibrosarcoma protuberans, B) Coverage defect measuring 353 cm², omega-shaped flap measuring 589 cm², (yellow marking represents the pedicular area - 15%), C) Six-month follow-up.

Several technical strategies were identified as contributors to improved flap advancement and successful defect coverage. These included designing flaps larger than the defect, positioning the pedicular area strategically relative to the defect, and performing subfascial dissection to maximize mobility. Together, these strategies facilitated adequate defect coverage while preserving flap perfusion, even in complex reconstructive scenarios. (Fig. 4)

Compared to alternative reconstructive options, including musculocutaneous or free flaps, freestyle multiple-perforator island flaps may offer advantages in selected patients by reducing operative time, avoiding donor-site functional morbidity, and eliminating the need for microsurgical resources. However, these findings should be interpreted with caution, as this study was not designed to perform direct comparisons between techniques.

This study has several limitations. Its retrospective and non-comparative design, along with the relatively small sample size, limits the strength of causal inferences and generalizability. Additionally, the absence of a control group and the potential for selection and information bias must be acknowledged. Despite these limitations, the inclusion of consecutive patients, the use of a standardized surgical approach, and consistent postoperative follow-up provide a reliable descriptive overview of outcomes in this cohort.

## Conclusion

In this retrospective series, freestyle multiple-perforator island flaps provided reliable coverage for large dorsolumbar defects, with a low rate of complications and no cases of total flap loss. Flap viability was preserved even with relatively small pedicular areas, supporting greater flexibility in flap design. These findings suggest that freestyle multiple-perforator island flaps represent a practical locoregional reconstructive option in selected patients. Further comparative studies are required to validate these observations and better define their role relative to other reconstructive techniques.

## Ethical approval

Ethical approval for this study was obtained from the Ethics Committee of the Faculty of Medicine at Fundación Universitaria Sanitas (Approval No CEIFUS 1581–25; Protocol No 088–25 UNV). The study was conducted in accordance with the principles of the Declaration of Helsinki.

## Patient consent

Informed consent was obtained from all patients for the use of clinical photographs.

## Declaration of AI and AI-assisted technologies in the writing process

The authors used ChatGPT solely for language translation and editing assistance. All scientific content, interpretation, and final manuscript responsibility remain with the authors.

## Declaration of competing interest

The authors declare that they have no known competing financial interests or personal relationships that could have appeared to influence the work reported in this paper.
